# Dim Range-Spread Target Detection for Stepped-Frequency Radar Using a Bernoulli Extended Target Filter

**DOI:** 10.3390/s25051426

**Published:** 2025-02-26

**Authors:** Fei Cai, Meiyu Tang

**Affiliations:** College of Information Science and Engineering, Hunan Normal University, Changsha 410081, China; meiyutang@hunnu.edu.cn

**Keywords:** extended target, target detection, stepped-frequency radar

## Abstract

Stepped-frequency radar is an important high range resolution radar. It can achieve wide overall bandwidth with narrow instant bandwidth. When the signal-to-noise ratio is low, detection and tracking become challenging due to dense false alarms and the range-Doppler coupling problem. In this paper, a new methodology is presented to address this problem. A Bernoulli extended target filter is used for joint detection and tracking of a dim range-spread target. The results of coherent processing are thresholded firstly using a low threshold, and the range-Doppler coupled detections generated by multiple scatterers, together with the false alarms, are fed to the Bernoulli filter. By appropriately modeling the range-Doppler coupling, the range spread, and the false alarms in the state and measurement models, the filter can detect the target effectively. Simulation results show that good detection performance is obtained and the range-Doppler coupling is decoupled simultaneously.

## 1. Introduction

The stepped-frequency waveform is a typical high range resolution (HRR) waveform, widely used in modern radar systems to achieve wide or ultra-wide bandwidth [1,2,3]. It offers high range resolution while retaining the advantages of lower instantaneous receiver bandwidth and a lower analog-to-digital sampling rate. The disadvantages of this waveform include the complications caused by range-Doppler coupling due to target motion. This results in target range shift and spreading of the target return [4]. The general solution to this problem is to apply velocity compensation to the received signal. However, this is not an easy task because targets are usually uncooperative, and the stepped-frequency waveform does not provide a direct target velocity measurement [5]. To resolve the range-Doppler coupling, one solution is to insert another batch of pulses with the opposite frequency slope or no frequency slope [3]. However, when there are multiple targets or scatterers, associating them may become difficult. Radial velocity estimation and range shift compensation for stepped-frequency waveforms have received extensive attention [6].

With the development of stealth technology and unmanned aerial vehicles (UAVs), more and more targets are of low observability. Due to range-Doppler coupling, range cell migration is a serious issue for stepped-frequency waveforms. As a result, noncoherent or coherent integration between frames becomes difficult. Furthermore, when the signal-to-noise ratio (SNR) is low, the detection threshold must be set low enough to ensure that the target is detected. However, this also results in many false alarms being generated, which further complicates the association process.

Detection and tracking of extended targets has received increasing research attention over the past decades [7,8,9,10,11,12,13,14,15,16]. For joint detection and tracking of a single extended target, Ristic developed a Bernoulli filter using a binomial random finite set (RFS), which is called BPF-X [17]. In [18], we proposed a Bernoulli filter for a single extended target. This filter models the extended target using a Poisson model, and it is optimal and computationally efficient. The Bernoulli filter is successfully used in radar dim target detection using track-before-detect (TBD) technology [19,20,21]. Unlike conventional detection and tracking algorithms, TBD can achieve superior performance using unthresholded data or data thresholded with significantly lower thresholds than those used in conventional trackers [22]. The methods in [19,20,21] all utilize unthresholded measurements. In fact, a Bernoulli filter that uses thresholded measurements can also be classified as a TBD method if the threshold is low enough. Hence, Bernoulli extended target filters [17,18] can also be used for joint detection and tracking of dim extended targets. Other TBD technologies such as the dynamic programming based TBD (DP-TBD) are also considered in radar systems [23,24]. The Bernoulli filter is generally implemented using a particle filter to handle non-linear and non-Gaussian problems. The particle filter algorithm is also an active research topic [25]. The field of radar and sonar has witnessed rapid development, and numerous research achievements have emerged in recent years [26,27,28].

In this paper, based on the Bernoulli filter in [18], we develop a joint detection and tracking filter for a range-spread target in stepped-frequency radar. Firstly, the results of coherent processing are thresholded using a low threshold, and the range-Doppler coupled detections generated by multiple scatterers, along with the false alarms, are fed to the Bernoulli filter. The range-Doppler coupling is modeled within the measurement model. The range-spread target is modeled as an extended target using a Poisson model. Numerical results show that the detection performance is greatly improved and that the range-Doppler coupling is decoupled.

The main contributions of this article are summarized as follows.

1. The challenging problem of dim extended target detection in stepped-frequency radar is studied. The Bernoulli extended target filter is introduced to address this issue. Bayesian modeling for the target and measurements is constructed. The Bernoulli extended target filter, along with its particle filter implementation, is designed.
2. Performance evaluation results, compared with benchmark methods, are presented. The target buried in noise can be reliably detected, and the detection performance is remarkably improved compared to classical methods. Meanwhile, the range and velocity of the target are decoupled.

The rest of this article is organized as follows. The signal model is presented in Section 2. The Bayesian modeling is presented in Section 3. The Bernoulli extended target filter and its approximated implementation are presented in Section 4. Simulation results are presented in Section 5. Conclusions are presented in Section 6.

## 2. Problem Formulation

We consider the conventional stepped-frequency signal, where the carrier frequency of successive pulses is increased by a constant offset, and the base waveform is a simple constant-frequency pulse. In this case, the transmitted signal within a frame is

(1) $$s\left(t\right)=\sum_{i=0}^{N_{p}-1}\mathrm{rect}\left(\frac{t-iT_{r}-\tau /2}{\tau }\right)\exp\left(j2\pi \left(f_{0}+i\Delta f\right)t\right)$$

where $T_{r}$ is the pulse repetition period, τ is the pulse width, $f_{0}$ is the carrier frequency of the first pulse, Δf is the fixed frequency step, $N_{p}$ is the number of pulses in a frame, *c* is the speed of light, and rect(·) is a rectangular window.

The local-oscillator signal is

(2) $$z\left(t\right)=\sum_{i=0}^{N_{p}-1}\mathrm{rect}\left(\frac{t-iT_{r}-T_{r}/2}{T_{r}}\right)\exp\left(j2\pi \left(f_{0}+i\Delta f\right)t\right)$$

The echo from a stationary scatterer at range *R* is given by

(3) $$\begin{array}{cc} r_{RF}\left(t\right)= & \sum_{i=0}^{N_{p}-1}\mathrm{rect}\left(\frac{t-iT_{r}-\tau /2-2R/c}{\tau }\right)\\ & \times \exp\left(j2\pi \left(f_{0}+i\Delta f\right)\left(t-2R/c\right)\right)+w_{RF}\left(t\right) \end{array}$$

where $w_{RF}\left(t\right)$ is the receiver noise at the radio frequency (RF) stage.

After mixing with the local-oscillator signal, the baseband signal is

(4) $$\begin{array}{cc} r_{BS}\left(t\right)= & \sum_{i=0}^{N_{p}-1}\mathrm{rect}\left(\frac{t-iT_{r}-\tau /2-2R/c}{\tau }\right)\\ & \times \exp\left(j4\pi \left(f_{0}+i\Delta f\right)R/c\right)+w_{BS}\left(t\right) \end{array}$$

where $w_{BS}\left(t\right)$ is the receiver noise at the baseband.

The baseband signal is sampled at time instant $iT_{r}+mT_{s}$ for the *i*th pulse, with m=0,1,⋯,M−1; $T_{s}$ is the sample interval, and *M* is the number of samples in each pulse. For a frame, there are in total $N_{p}M$ samples, which can be rearranged as an $N_{p}\times M$ data matrix.

The IDFT is then applied to each column of the data matrix to obtain the intensities of the echo at the effective range bins. This process is known as stretch processing. The result of stretch processing is an $N_{p}\times M$ data matrix (if the IFFT is used, the number of rows may be a power of 2 larger than $N_{p}$), which is denoted as R. Note that for each sampling point, the IDFT result comprises $N_{p}$ bins, with the *i*th bin corresponding to the range $(i+aN_{p})\Delta R$. Here, $\Delta R=c/(2N_{p}\Delta f)$ represents the range resolution, and *a* is the ambiguity number. The ambiguity number *a* can be determined using the column index *m*. If, for each pulse, the *m*th sampling point has sampled the echo signal from the scatterer, such that $2R/c\leq \left(m-1\right)T_{s}<2R/c+\tau$, then we can obtain the high-resolution range profile (HRRP) of the target from the *m*th column of R. A peak will appear at the (i,m)th element of R, where $i=\mathrm{mod}(\mathrm{round}\left(R/\Delta R\right),N_{p})$, and the range information of the scatterer can be deduced from the index (i,m).

If the scatterer has a radial velocity *v* (where v>0 denotes that the target is departing from the radar, and vice versa) and its range at t=0 is $R_{0}$, then its range at *t* is $R\left(t\right)=R_{0}+vt$. The phase in (4) becomes

(5) $$\begin{array}{cc} \phi (i) & =\frac{4\pi }{c}\left(f_{0}+i\Delta f\right)\left(R_{0}+vt_{i}\right)\\ & \approx \frac{4\pi }{c}\left(f_{0}+i\Delta f\right)\left(R_{0}+vT_{r}i\right)\\ & =\frac{4\pi }{c}\left(f_{0}R_{0}+\Delta fR_{0}i+f_{0}vT_{r}i+\Delta fvT_{r}i^{2}\right) \end{array}$$

From (5) we observe that the third term induces movement of the peak, while the fourth term leads to distortion of the peak. The third term gives rise to range-Doppler coupling, and the range shift associated with target velocity is expressed as $f_{0}vT_{r}/\Delta f$.

If there are $N_{s}$ stationary scatterers, then the echo signal is given by

(6) $$\begin{array}{cc} r_{RF}\left(t\right)= & \sum_{i=0}^{N_{p}-1}\sum_{s=1}^{N_{s}}\mathrm{rect}\left(\frac{t-iT_{r}-\tau /2-2R_{s}/c}{\tau }\right)\\ & \times \exp\left(j2\pi \left(f_{0}+i\Delta f\right)\left(t-2R_{s}/c\right)\right)+w_{RF}\left(t\right) \end{array}$$

where $R_{s}$ is the range of the *s*th scatterer. The corresponding signals at the following stages can be derived similarly.

**Figure 1:**
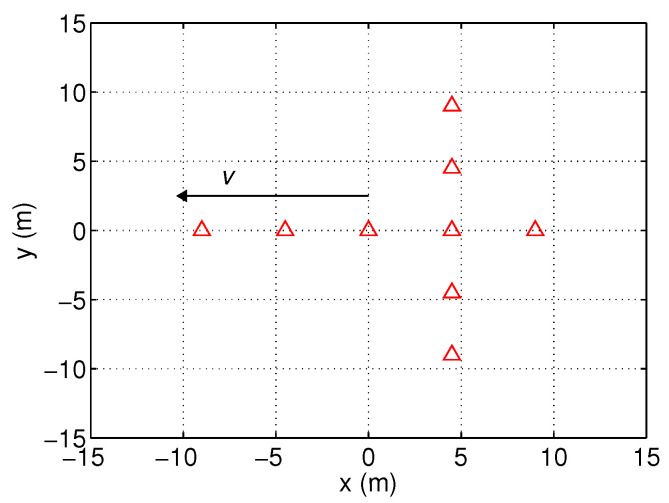
Illustration of target scatterers.

In this article, we consider an extended target consisting of $N_{s}$ scatterers, where the distances between them are not negligible compared with the range resolution. Hence, there will be multiple peaks in the HRRP. Similar to that in [29], an extended target as shown in Figure 1 is considered, which is an aircraft containing nine scatterers. The target scatterer model is assumed to be known in this article.

## 3. Bayesian Modeling

We assume that there is at most one target in the scene, and the target can be absent or present. The state vector of the target is denoted as $x={\left(R,v\right)}^{T}$, where *R* and *v* are the range and relative velocity of the target centroid, respectively. Using the Finite Set Statistics (FISST), the target state can be modeled as a Bernoulli random finite set (RFS) *X*. If the target is present, then X={x}; otherwise, if the target is absent, then X=∅. The probability density of the RFS *X* at time step *k* is given by

(7) $$f_{k|k}\left(X\right)=\left\{\begin{array}{cc} 1-p_{k|k} & \mathrm{if}X=\emptyset \\ p_{k|k}f_{k|k}\left(x\right) & \mathrm{if}X=\{x\} \end{array}\right.$$

where $f_{k|k}\left(x\right)$ is the probability density of the target state and $p_{k|k}$ is the probability of target existence.

### 3.1. Target Dynamic Model

Target motion is modeled as a nearly constant velocity model

(8) $$x_{k}=Fx_{k-1}+w_{k}$$

where F is the transition matrix and $w_{k}∼N\left(0,Q\right)$ is the white Gaussian process noise. We adopt

(9) $$\begin{array}{cc} F & =\left(\begin{array}{cc} 1 & T\\ 0 & 1 \end{array}\right) \end{array}$$

(10) $$\begin{array}{cc} Q & =\sigma_{w}^{2}\left(\begin{array}{cc} \frac{1}{4}T^{4} & \frac{1}{2}T^{3}\\ \frac{1}{2}T^{3} & T^{2} \end{array}\right) \end{array}$$

where *T* is the interval between the time steps and $\sigma_{w}^{2}$ is the variance of the process noise.

We model the target presence by a first-order two-state Markov chain. If the target is not present at time step k−1, we assume that it may enter the scene at time step *k* with probability $p_{B}$. The density of the newborn target is called the birth density and is denoted as $b_{k|k-1}\left(x\right)$. In this article, the birth density is adopted as the uniform density over the state space of interest. In this case, the Markov density of the Bernoulli RFS *X* is given by

(11) $$f_{k|k-1}\left(X|\emptyset \right)=\left\{\begin{array}{cc} 1-p_{B} & \mathrm{if}X=\emptyset \\ p_{B}b_{k|k-1}\left(x\right) & \mathrm{if}X=\{x\} \end{array}\right.$$

If a target with state $x^{\prime }$ is present at time step k−1, we assume that it can survive to time step *k* with probability $p_{S}$. The dynamic between time steps k−1 and *k* is modeled by the Markov density $f_{k|k-1}\left(x|x^{\prime }\right)$. According to (8), we have $f_{k|k-1}\left(x|x^{\prime }\right)=N\left(x;Fx^{\prime },Q\right)$. Then, the Markov density of the Bernoulli RFS *X* is given by

(12) $$f_{k|k-1}\left(X|\left\{x^{\prime }\right\}\right)=\left\{\begin{array}{cc} 1-p_{S} & \mathrm{if}X=\emptyset \\ p_{S}f_{k|k-1}\left(x|x^{\prime }\right) & \mathrm{if}X=\{x\} \end{array}\right.$$

### 3.2. Measurement Model

The result of the stretch processing is thresholded with a comparatively low threshold to ensure that the scatterers of the target can be detected with a high probability of detection ($p_{D}$). Since we have assumed that the target contains multiple scatterers and the range resolution of the radar can resolve them, multiple detections may be generated by the target. Due to the low detection threshold, a lot of false alarms are generated by the detector at the same time. The collection of all the indices of those bins in R that pass the threshold is used as the measurement set. The measurement set *Z* at each frame is obtained by

(13) $$\begin{array}{cc} Z={\left\{z_{j}\right\}}_{j=1}^{N_{z}}= & \{\left(i,m\right)||R_{i,m}|>\tau_{1},i=0,\cdots ,N_{p}-1,\\ & m=0,\cdots ,M-1\} \end{array}$$

where $z_{j}$ is the *j*th measurement, $\tau_{1}$ is the primary threshold to give a certain primary probability of false alarm $p_{F}$, and $N_{z}=\left|Z\right|$ is the number of measurements in the set *Z*.

The detections *Z* in (13) are the union of the target-generated detections and the false alarms. We denote the set of target-generated detections as *W*. Then, the set of false alarms can be expressed as K=Z−W.

As in [18], we model the set of target-generated measurements as a Poisson model proposed by [7]. The number of the target-generated measurements is assumed to follow a Poisson distribution with mean γ(x). The measurements from the target are distributed according to the spatial extent model $\phi_{x}\left(z\right)$. Then, the likelihood function of the extended target is

(14) $$L_{W}\left(x\right)≜f\left(W|x\right)=e^{-\gamma (x)}\prod_{z\in W}\gamma \left(x\right)\phi_{x}\left(z\right)$$

This spatial distribution function could be a bounded distribution, such as a uniform pdf, or an unbounded distribution, such as a Gaussian. In this case, the uniform distribution is selected, and the spatial extent model $\phi_{x}\left(z\right)$ is defined as

(15) $$\phi_{x}\left(z=\left(i,m\right)\right)=\left\{\begin{array}{cc} 1/L_{r} & \begin{array}{c} l\mathrm{if}|i\Delta R-R-f_{0}{\mathrm{vT}}_{r}/\Delta f|<L_{r}/2\\ \mathrm{and}2R/c\leq \left(m-1\right)T_{s}<2R/c+\tau \end{array}\\ 0 & \mathrm{if}\mathrm{otherwise} \end{array}\right.$$

where $L_{r}$ is the maximum target extent. As the target scatterer model is assumed to be known in this article, $L_{r}$ can be determined in advance. We also assume that γ(x) is a known constant, i.e., γ(x)=γ. As the Poisson model has been used to model the number of target-generated measurements, the probability that the target generates no measurement (i.e., |W|=0) is $e^{-\gamma }$. Hence, the probability that the target generates at least one measurement is $1-e^{-\gamma }$. As a result, denoting the probability of detecting the target as a whole as $p_{D}\left(x\right)$, the probability that at least one target-generated detection exists is $p_{D}^{\prime }\left(x\right)≜\left(1-e^{-\gamma }\right)p_{D}\left(x\right)$. Assume the probability of detection for each scatterer is $p_{D}^{\left(i\right)},i=1,\ldots ,N_{s}$, then the probability of detection as a whole is $p_{D}\left(x\right)=1-\prod_{i=1}^{N_{s}}\left(1-p_{D}^{\left(i\right)}\right)$.

The false alarm process in this case can be modeled as a Poisson process ([30], Section 9.3), i.e.,

(16) $$\kappa \left(K\right)=e^{-\lambda_{C}}\prod_{z\in K}\lambda_{C}c\left(z\right)$$

where c(z) is the probability density of the false alarms, and $\lambda_{C}$ is the mean number of the false alarms. The number of false alarms follows a Poisson distribution with the parameter $\lambda_{C}$. Assume the probability of false alarm of all the bins in the threshold detection is $p_{F}$, then $\lambda_{C}=N_{p}Mp_{F}$. A homogeneous noise background is assumed and then the false alarms are uniformly distributed over the sensor field-of-view. Thus, we have $c\left(z\right)=1/\left(N_{p}M\Delta R\right)$. Then, (16) can be simplified as

(17) $$\kappa \left(K\right)=e^{-N_{p}MP_{F}}{\left(p_{F}/\Delta R\right)}^{\left|K\right|}$$

Strictly speaking, the false alarm process is not independent of the target-observation process in this case. If the target is present, some of the bins are occupied by the target, resulting in a mean number of false alarms $\lambda_{C}$ that is less than $N_{p}Mp_{F}$. However, we neglect this and assume they are independent in our implementation.

As *X* is a Bernoulli RFS, the likelihood function f(Z|X) takes two forms, one for X=∅ and the other for X={x}, respectively. Using the convolution formula from the FISST calculus ([30], Section 11.5), we can determine that the likelihood function f(Z|X) is given by

(18) $$\begin{array}{cc} f(Z|X) & =\sum_{W\subseteq Z}f\left(W|X\right)\kappa \left(Z-W\right)\\ & =f\left(\emptyset |X\right)\kappa \left(Z\right)+\sum_{W\subseteq Z}f\left(W|X\right)\kappa \left(Z-W\right) \end{array}$$

where *W* is nonempty and the time subscript *k* has been omitted for convenience. For the case X=∅, we have

(19) $$\begin{array}{cc} f(Z|\emptyset ) & =f\left(\emptyset |\emptyset \right)\kappa \left(Z\right)+\sum_{W\subseteq Z}f\left(W|\emptyset \right)\kappa \left(Z-W\right)\\ & =1\cdot \kappa \left(Z\right)+\sum_{W\subseteq Z}0\cdot \kappa \left(Z-W\right)=\kappa \left(Z\right) \end{array}$$

For the case X={x}, we have

(20) $$\begin{array}{c} f(Z|\{x\})\\ =f\left(\emptyset |\left\{x\right\}\right)\kappa \left(Z\right)+\sum_{W\subseteq Z}f\left(W|\left\{x\right\}\right)\kappa \left(Z-W\right)\\ =\left(1-p_{D}^{\prime }\left(x\right)\right)\kappa \left(Z\right)+p_{D}\left(x\right)\sum_{W\subseteq Z}L_{W}\left(x\right)\kappa \left(Z-W\right)\\ =\kappa \left(Z\right)\left(1-p_{D}^{\prime }\left(x\right)+p_{D}\left(x\right)\sum_{W\subseteq Z}L_{W}\left(x\right)\frac{\kappa (Z-W)}{\kappa (Z)}\right) \end{array}$$

## 4. The Bernoulli Extended Target Filter and Its Approximated Implementation

### 4.1. The Bernoulli Extended Target Filter

The Bernoulli filter is the optimal recursive Bayesian estimator for joint detection and tracking of a single target. In this filter, the posterior distributions of the random finite $f_{k|k}\left(X\right)$ are propagated over time. These distributions include two quantities, as shown in (7): the first is the posterior probability of target existence, denoted as $p_{k|k}$, and the second is the target’s track density, denoted as $f_{k|k}\left(x\right)$. In each time step *k*, given $p_{k-1|k-1}$ and $f_{k-1|k-1}\left(x\right)$, $p_{k|k}$ and $f_{k|k}\left(x\right)$ are recursively computed using two steps: prediction and correction. After that, using the computed $p_{k|k}$ and $f_{k|k}\left(x\right)$, target detection can be performed by thresholding $p_{k|k}$, and the target state can be estimated using $f_{k|k}\left(x\right)$.

In this article, the range-spread target is modeled as an extended target using a Poisson extended target model. The predictor and corrector of the Bernoulli extended target filter for this model are given as follows. The detailed derivation can be found in [18].

*Predictor:* Given $p_{k-1|k-1}$ and $f_{k-1|k-1}\left(x\right)$, the $p_{k|k-1}$ and $f_{k|k-1}\left(x\right)$ are predicted, respectively, as

(21) $$p_{k|k-1}=p_{B}\left(1-p_{k-1|k-1}\right)+p_{S}p_{k-1|k-1}$$

and

(22) $$\begin{array}{c} \;\\ f_{k|k-1}\left(x\right)=\frac{p_{B}b_{k|k-1}\left(x\right)\left(1-p_{k-1|k-1}\right)}{p_{k|k-1}}\\ +\frac{p_{S}p_{k-1|k-1}\int f_{k|k-1}\left(x|x^{\prime }\right)f_{k-1|k-1}\left(x^{\prime }\right)dx^{\prime }}{p_{k|k-1}} \end{array}$$

*Corrector:* Given $p_{k|k-1}$ and $f_{k|k-1}\left(x\right)$, the $p_{k|k}$ and $f_{k|k}\left(x\right)$ are updated, respectively, according to

(23) $$\begin{array}{cc} p_{k|k}\; & =\frac{p_{k|k-1}\int l_{k}\left(Z|x\right)f_{k|k-1}\left(x\right)dx}{1-p_{k|k-1}+p_{k|k-1}\int l_{k}\left(Z|x\right)f_{k|k-1}\left(x\right)dx} \end{array}$$

(24) $$\begin{array}{cc} f_{k|k}\left(x\right)\; & =\frac{l_{k}\left(Z|x\right)f_{k|k-1}\left(x\right)}{\int l_{k}\left(Z|x\right)f_{k|k-1}\left(x\right)dx} \end{array}$$

where

(25) $$\begin{array}{cc} l_{k}\left(Z|x\right) & ≜\frac{f(Z_{k}|\left\{x_{k}\right\})}{f(Z_{k}|\emptyset )}\\ & =1-p_{D}^{\prime }\left(x_{k}\right)+p_{D}\left(x_{k}\right)\sum_{W\subseteq Z_{k}}\frac{L_{W}\left(x_{k}\right)\kappa \left(Z_{k}-W\right)}{\kappa (Z_{k})} \end{array}$$

### 4.2. Particle Filter Implementation

We choose to implement the Bernoulli extended target filter using the sequential Monte Carlo (SMC) method. Particles ${\left\{x_{k}^{\left(n\right)},w_{k}^{\left(n\right)}\right\}}_{n=1}^{N}$ are utilized to approximate the Bayes posterior $f_{k|k}\left(x\right)$. Here, *N* is the number of existing particles, $x_{k}^{\left(n\right)}$ represents the state vector of the *n*th particle, and $w_{k}^{\left(n\right)}$ is the corresponding weight. The weights ${\left\{w_{k}^{\left(n\right)}\right\}}_{n=1}^{N}$ are normalized to ensure that $\sum_{n=1}^{N}w_{k}^{\left(n\right)}=1$.

*Predictor:* The predicted probability of existence $p_{k|k-1}$ is first computed using (21). The density $f_{k|k-1}\left(x\right)$ is approximated as

(26) $$f_{k|k-1}\left(x\right)\approx \sum_{n}^{N+B}w_{k|k-1}^{\left(n\right)}\delta_{x_{k|k-1}^{\left(n\right)}}\left(x\right)$$

where the particles are drawn from two proposal distributions:

(27) $$x_{k|k-1}^{\left(n\right)}∼\left\{\begin{array}{cc} f_{k|k-1}\left(x|x_{k-1}^{\left(n\right)}\right) & n=1,\cdots ,N\\ b_{k|k-1}\left(x\right) & n=N+1,\cdots ,N+B \end{array}\right.$$

with weights

(28) $$w_{k|k-1}^{\left(n\right)}=\left\{\begin{array}{cc} \frac{p_{S}p_{k-1|k-1}}{Np_{k|k-1}} & n=1,\cdots ,N\\ \frac{p_{B}\left(1-p_{k-1|k-1}\right)}{Bp_{k|k-1}} & n=N+1,\cdots ,N+B \end{array}\right.$$

where *B* is the number of birth particles. To reduce computational complexity, compute ${w^{\prime }}_{k|k-1}^{\left(n\right)}$ instead:

(29) $${w^{\prime }}_{k|k-1}^{\left(n\right)}=\left\{\begin{array}{cc} p_{s}p_{k-1|k-1}/N & n=1,\cdots ,N\\ p_{b}\left(1-p_{k-1|k-1}\right)/B & n=N+1,\cdots ,N+B \end{array}\right.$$

*Corrector:* The integral features in (23) and (24) are approximated by

(30) $$\int l_{k}\left(Z|x\right)f_{k|k-1}\left(x\right)dx\approx \sum_{n=1}^{N+B}l_{k}\left(Z|x_{k|k-1}^{\left(n\right)}\right)w_{k|k-1}^{\left(n\right)}$$

Consequently, (23) can be approximated by

(31) $$\begin{array}{c} p_{k|k}\approx \frac{p_{k|k-1}\sum_{n=1}^{N+B}l_{k}\left(Z|x_{k|k-1}^{\left(n\right)}\right)w_{k|k-1}^{\left(n\right)}}{1-p_{k|k-1}+p_{k|k-1}\sum_{n=1}^{N+B}l_{k}\left(Z|x_{k|k-1}^{\left(n\right)}\right)w_{k|k-1}^{\left(n\right)}}\\ =\frac{\sum_{n=1}^{N+B}l_{k}\left(Z|x_{k|k-1}^{\left(n\right)}\right){w^{\prime }}_{k|k-1}^{\left(n\right)}}{1-p_{k|k-1}+\sum_{n=1}^{N+B}l_{k}\left(Z|x_{k|k-1}^{\left(n\right)}\right){w^{\prime }}_{k|k-1}^{\left(n\right)}} \end{array}$$

and (24) can be approximated by

(32) $$\begin{array}{cc} f_{k|k}\left(x\right) & \approx \frac{\sum_{n=1}^{N+B}l_{k}\left(Z|x_{k|k-1}^{\left(n\right)}\right)w_{k|k-1}^{\left(n\right)}\delta_{x_{k|k-1}^{\left(n\right)}}\left(x\right)}{\sum_{n=1}^{N+B}l_{k}\left(Z|x_{k|k-1}^{\left(n\right)}\right)w_{k|k-1}^{\left(n\right)}}\\ & =\frac{\sum_{n=1}^{N+B}l_{k}\left(Z|x_{k|k-1}^{\left(n\right)}\right){w^{\prime }}_{k|k-1}^{\left(n\right)}\delta_{x_{k|k-1}^{\left(n\right)}}\left(x\right)}{\sum_{n=1}^{N+B}l_{k}\left(Z|x_{k|k-1}^{\left(n\right)}\right){w^{\prime }}_{k|k-1}^{\left(n\right)}} \end{array}$$

From (31) and (32) we see that ${w^{\prime }}_{k|k-1}^{\left(n\right)}$ is sufficient for the filtering problem, and the updated weight is

(33) $$w_{k}^{\left(n\right)}=\frac{l_{k}\left(Z|x_{k|k-1}^{\left(n\right)}\right){w^{\prime }}_{k|k-1}^{\left(n\right)}}{\sum_{n=1}^{N+B}l_{k}\left(Z|x_{k|k-1}^{\left(n\right)}\right){w^{\prime }}_{k|k-1}^{\left(n\right)}}$$

Note that $w_{k}^{\left(n\right)}\propto {\tilde{w}}_{k}^{\left(n\right)}≜l_{k}\left(Z|x_{k|k-1}^{\left(n\right)}\right){w^{\prime }}_{k|k-1}^{\left(n\right)}$. Hence, our first step is to obtain ${\left\{{\tilde{w}}_{k}^{\left(n\right)}\right\}}_{n=1}^{N+B}$ in order to update $p_{k|k}$. Subsequently, we normalize these weights to obtain ${\left\{w_{k}^{\left(n\right)}\right\}}_{n=1}^{N+B}$, with

(34) $$\begin{array}{c} l_{k}\left(Z|x_{k|k-1}^{\left(n\right)}\right)\\ =\frac{f(Z_{k}|\left\{x_{k|k-1}^{\left(n\right)}\right\})}{f(Z_{k}|\emptyset )}\\ =1-p_{D}^{\prime }\left(x_{k|k-1}^{\left(n\right)}\right)+p_{D}\left(x_{k|k-1}^{\left(n\right)}\right)\\ \times \sum_{W\subseteq Z_{k}}\frac{L_{W}\left(x_{k|k-1}^{\left(n\right)}\right)\kappa \left(Z_{k}-W\right)}{\kappa (Z_{k})} \end{array}$$

In [18], it is derived that

(35) $$\begin{array}{c} \sum_{W\subseteq Z}\frac{L_{W}\left(x_{k|k-1}^{\left(n\right)}\right)\kappa \left(Z_{k}-W\right)}{\kappa (Z_{k})}\\ =e^{-\gamma }\left(\prod_{j=1}^{|Z_{k}|}\left(1+\frac{\gamma \phi_{x_{k|k-1}^{\left(n\right)}}\left(z_{j}\right)}{\lambda_{C}c\left(z_{j}\right)}\right)-1\right) \end{array}$$

Then, we can obtain

(36) $$\begin{array}{c} l_{k}\left(Z|x_{k|k-1}^{\left(n\right)}\right)\\ =1-p_{D}^{\prime }\left(x_{k|k-1}^{\left(n\right)}\right)+p_{D}\left(x_{k|k-1}^{\left(n\right)}\right)\\ \times e^{-\gamma }\left(\prod_{j=1}^{|Z_{k}|}\left(1+\frac{\gamma \Delta R\phi_{x_{k|k-1}^{\left(n\right)}}\left(z_{j}\right)}{p_{F}}\right)-1\right) \end{array}$$

*Estimator:* Target detection can be performed using $p_{k|k}$. If $p_{k|k}$ exceeds the predefined threshold τ∈(0,1), target presence is declared. If a target is detected, the target state is estimated using the particles before resampling by the minimum mean-square error (MMSE) estimator:

(37) $${\hat{x}}_{k|k}=\sum_{n=1}^{N+B}w_{k}^{\left(n\right)}x_{k|k-1}^{\left(n\right)}$$

## 5. Numerical Study

In this section, we analyze the performance of the proposed algorithm through Monte Carlo simulations. For comparison, the BPF-X in [17] is also adapted to this problem. As shown in Figure 1, the extended target is represented by nine scatterers, i.e., $N_{s}=9$. For simplicity, the echo energies of all the scatterers are assumed to be equal and time invariant. The SNR for a single scatterer is 6 dB after IFFT. The radar is assumed to be stationary and located at the origin of the axes. The target is introduced at frame 11 and deleted at frame 51.

The radar parameters are shown in Table 1. Then, we can determine that the number of samples in a pulse is $M=T_{r}/T_{s}=10$, and the range resolution is $\Delta R=c/(2N_{p}\Delta f)=0.3906$ m. Hence, the distances between the target scatterers are large compared with the range resolution. In general, they can be resolved; however, in some cases, more than one scatterer may be located within a single range bin (i.e., they may be unresolved).

**Figure 2:**
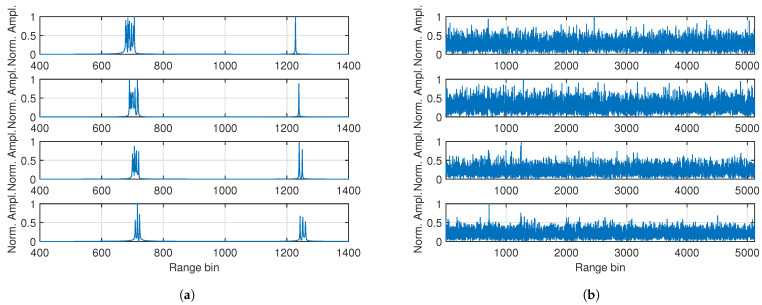
Normalized amplitudes of the rearranged results of the stretch processing in frames 11, 21, 31, and 41 (in order from top to bottom). (**a**) Part of the results, without background noise. (**b**) The entirety of the results, with background noise.

For the convenience of display, the $N_{p}\times M$ data matrix after stretch processing is rearranged as a data vector with length $N_{p}M=5200$. The resulting data vectors from a single run, with and without background noise, are shown in Figure 2a,b, respectively. From Figure 2a we can see that several peaks are generated by the scatterers of the target. Hence, the target can be considered as a range-spread target or an extended target. In the four frames, the peaks are separated into two groups. The reason for this is that some of the scatterers are located in the next sample from the others due to the range differences between them. In Figure 2b, the peaks generated by the target are submerged by the background noises, making it difficult to determine the target’s existence and location.

**Figure 3:**
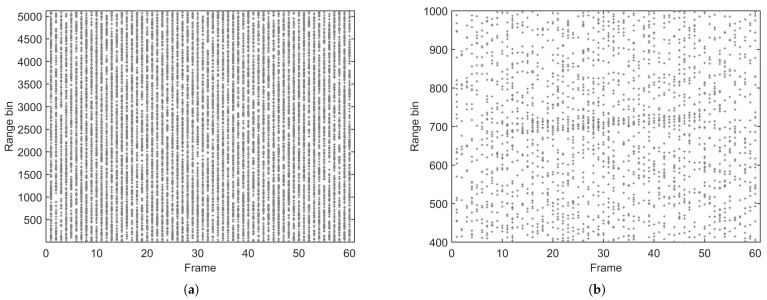
Detections of a single run, with $P_{F1}$=0.05. (**a**) All results. (**b**) Local details.

We set the detection threshold to achieve a false alarm probability $p_{F}=0.05$. The corresponding probability of detection for each scatterer is identical and takes the value $p_{D}^{\left(i\right)}=0.72$, for $i=1,\cdots ,N_{s}$. The detection results from one run are shown in Figure 3. We can observe that the detections generated by the extended target are submerged among the false detections.

According to the target scatterer model, the maximum target extent is set to $L_{r}=20$ m. The mean number of target measurements γ(x) for the proposed filter should be determined by the known target scatterer model and the range resolution ΔR. Here, we set γ(x)=7.2 (i.e., $p_{D}^{\prime \prime }=0.72$ for the BPF-X). For the particle filter, each frame is considered as a time step, specifically $T=N_{p}T_{r}$. The particle filter parameters of the proposed filter and the BPF-X are the same and are set as follows: probability of target birth $p_{B}=0.05$, target survival probability $p_{S}=0.95$, initial target existence probability $p_{0|0}=0.1$, number of existing particles N=5000, and number of birth particles B=1000. The target range of interest lies within the interval $\left(\tau ,T_{r}\right)c/2=\left(60,600\right)$ m, and the Doppler velocity of interest is within the interval (−300,300) m/s. The initial and birth densities of *R* and *v* are uniformly distributed within their respective intervals of interest. The BPF-X is implemented with a gating threshold of η=10 (which results in an average $|Z_{k}^{*}|\approx 6.60$, and a maximum value of $|Z_{k}^{*}|=28$), and a fixed number of scatterers $L_{k}\equiv 9$ (this is the true value, not estimated here for the purpose of a fair comparison, and also because the estimated $L_{k}$ values tend to be negative).

**Figure 4:**
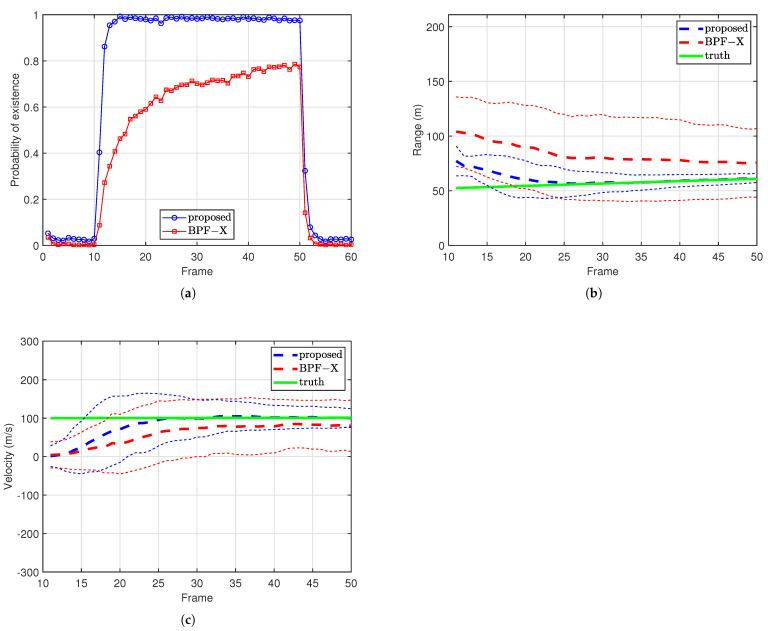
Filtering results. (**a**) Average probability of existence. (**b**) Range estimation results, with thin dashed lines representing the mean ± one standard deviation. (**c**) Velocity estimation results, with thin dashed lines indicating the mean ± one standard deviation.

Figure 4 shows the performance of the proposed method and the BPF-X in 200 Monte Carlo runs. Figure 4a shows the probability of existence averaged over 200 Monte Carlo runs. From Figure 4a we see that the target can be detected with a small delay using $p_{k|k}$ as the test statistic. Figure 4b,c shows the estimation performance of range and Doppler, respectively. We can see that the range-Doppler coupling is resolved and the estimations converge to the true values. The BPF-X exhibits worse performance in both detection and estimation.

To evaluate the final detection performance of the proposed filter, we implement the *M* out of *N* detector for high-resolution radar, as described in [9], using the same data for comparison. The window length *N* is selected as 20 m/ΔR= 49, and *M* is set to M=7. The *M* out of *N* detector is assumed to have knowledge of the target centroid, thus eliminating the need for window sliding (i.e., the window is directly placed over the target’s location). Detection performances of the three methods, based on 200 Monte Carlo runs, are presented in Table 2. The final probability of false alarm $p_{Ffinal}$ is calculated using frames where the target does not exist (frames 1–10 and 51–60). The final probability of detection $p_{Dfinal}$ for the three methods is calculated using frames where the target does exist (frames 11–50). As expected, the detection performance of the proposed filter surpasses that of the BPF-X. Notably, the proposed filter achieves a higher $p_{Dfinal}$ at a lower $p_{Ffinal}$ compared to the *M* out of *N* detector, even without assuming knowledge of the target centroid.

**Figure 5:**
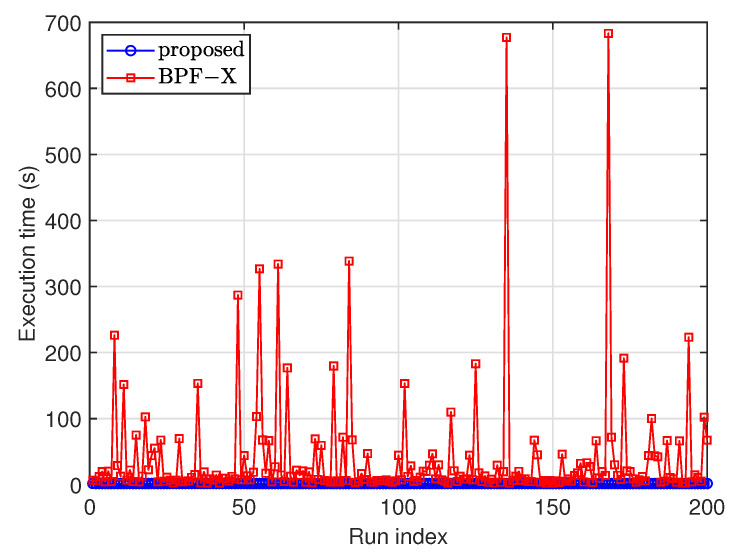
Execution time of each Monte Carlo run.

The execution time of each Monte Carlo run for the two filters is compared in Figure 5. All simulations were performed on a computer with an Intel i5-12450H CPU (produced by Intel Corporation, Santa Clara, CA, USA) and 16 GB of memory in the MATLAB 2023a environment. The execution time of the proposed filter was nearly constant, at approximately 1.61 s per run (or about 0.02 s on average for each frame), while the execution time of the BPF-X ranged from 2.42 s to 964.07 s. The computational complexity of the BPF-X is highly correlated with the number of gated measurements (i.e., $|Z_{k}^{*}|$). If $|Z_{k}^{*}|$ is occasionally large, the execution time will be significantly prolonged. For example, if $|Z_{k}^{*}|=28$, then the number of possible *W* in $P_{1:L_{k}}\left(Z_{k}^{*}\right)$ can be as large as ∑l=1Lk|Zk*|l>107. If the gating operation is not used, the BPF-X will become computationally intractable. If the proposed method is programmed in C language, it can run much faster than in MATLAB (in the order of a few tens times of faster) [31]. Hence, the proposed method is feasible for real-time applications. There are several ways to reduce the execution time of the proposed filter. First, reducing the particle number can decrease the execution time, but this may impact the detection and estimation performances, indicating a tradeoff between execution time and performance. Second, the parallelization of the particle filter can be utilized to meet runtime constraints without sacrificing performance [32]. There are many parallel computing processors available, such as multi-core CPUs, GPUs, multi-core DSPs, and FPGAs.

**Remark** **1.** *The detection and estimation performance of the BPF-X will be better if the gating threshold η is lower. However, the BPF-X is difficult to implement in that case. In fact, the BPF-X is practicable when the density of false alarms is comparatively low (as can be seen from the examples in [17]).*

## 6. Conclusions

In this article, we studied joint detection and tracking of a dim extended target using stepped-frequency radar. This problem has been addressed using a Bernoulli extended target filter. Firstly, the results after stretch processing are thresholded using a low threshold, and these detection results are then used as measurements by the Bernoulli filter. Range-Doppler coupling and range-spread are modeled in the state model and the measurement model of the filter. Numerical results demonstrate that the proposed filter can effectively detect and track the range-spread target with low SNR.

## Figures and Tables

**Table 1:** Radar parameters setup.

$f_{0}$	$N_{p}$	$T_{r}$	Δf	$T_{s}$	τ
35 GHz	512	4 μs	750 kHz	0.4 μs	0.4 μs

**Table 2:** Detection performance.

Parameters	Proposed	BPF-X	*M* out of *N*
$p_{Ffinal}$	0.0025	0.0025	0.0027
$p_{Dfinal}$	0.9654	0.6733	0.6761

## Data Availability

Data are contained within the article.
